# Monitoring of *RAS* mutant clones in plasma of patients with *RAS* mutant metastatic colorectal cancer

**DOI:** 10.1007/s12094-021-02767-7

**Published:** 2022-01-07

**Authors:** A. Fernández Montes, E. Élez, A. Vivancos, N. Martínez, P. González, M. Covela, J. de la Cámara, A. Cousillas, J. C. Méndez, B. Graña, E. Aranda

**Affiliations:** 1 Medical Oncology Department, Complexo Hospitalario Universitario de Ourense, Ourense, Spain; 2 Medical Oncology Department, Vall d’Hebron Barcelona Hospital Campus, Vall d’Hebron Institute of Oncology (VHIO), Universitat Autònoma de Barcelona, Barcelona, Spain; 3 Cancer Genomics Group, Vall d’Hebron Institute of Oncology (VHIO), Universitat Autònoma de Barcelona, Barcelona, Spain; 4 Medical Oncology Department, Hospital Universitario A Coruña, A Coruña, Spain; 5 Medical Oncology Department, Hospital Universitario Álvaro Cunqueiro, Vigo, Spain; 6 Medical Oncology Department, Hospital Universitario Lucus Augusti, Lugo, Spain; 7 Medical Oncology Department, Hospital Universitario Arquitecto Marcide, Ferrol, Spain; 8 Medical Oncology Department, Hospital Provincial de Pontevedra, Pontevedra, Spain; 9 Medical Oncology Department, Centro Oncológico de Galicia, A Coruña, Spain; 10 Medical Oncology Department, Hospital Universitario Reina Sofía, Córdoba, Spain

**Keywords:** Metastatic colorectal cancer, Circulating tumor DNA, Liquid biopsy, *RAS* mutant, EGFR inhibitors

## Abstract

**Purpose:**

Some patients with histologically confirmed primary mCRC and mutated *RAS* reported undetectable *RAS* mutant clones in plasma after receiving anti-VEGF treatment. The aim was to prospectively assess it with its potential therapeutic implications.

**Methods:**

*RAS* mutant genes in solid biopsy (before first-line treatment: FOLFOX/CAPOX + bevacizumab) were compared in liquid biopsy (before second-line treatment: panitumumab + FOLFIRI), using Idylla™ system. Discordant results between solid/liquid biopsies were assessed by the next-generation sequencing (NGS) test (solid/liquid biopsies).

**Results:**

Twenty-three patients were assessed (seven had *RAS* mutant discrepancies between solid/liquid biopsies). The NGS test confirmed that 3/23 (13%) patients had undetectable *RAS* mutant clones in liquid biopsy and 3/23 (13%) presented discrepancies in solid biopsy (Idylla™ system vs. NGS test).

**Conclusion:**

Thirteen percentage of patients had undetectable *RAS* mutant clones in liquid biopsy after first-line treatment. However, some discrepancies between solid and liquid biopsies have been observed. These results suggest a need to improve accuracy of *RAS* analyses, especially in solid biopsies.

## Introduction

Assessment of *RAS* mutations is crucial to guide treatment decisions in clinical practice. The determination must be performed in tumor tissue upon the diagnosis of metastatic disease either at the primary tumor or the metastatic disease [1].

Circulating cell-free tumor DNA (ctDNA) that originates from the currently present tumor has the same genetic and epigenetic alterations, which are related to tumor development, progression, and treatment resistance [2–4]. Moreover, ctDNA is more accurate than circulating tumor cells in respect of tumor burden and can be used as both a prognostic and diagnostic biomarker. It also acts as a predictor in the assessment of antineoplastic therapy through molecular analysis and mutation identification. *TP53* and *KRAS* mutations, microsatellite instability or loss of heterozygosity, together with DNA hyper-methylation can be detected using ctDNA [5]. The analysis of ctDNA also so-called as a “liquid biopsy” has been proposed as an alternative to the invasive techniques for obtaining tumor samples. This liquid biopsy enables minimally invasive monitoring of tumor evolution over, and could provide current genetic information before initiate second-line treatment [2–4].

Some patients with primary mCRC and *RAS* mutation have reported undetectable *RAS* mutant clones in plasma after receiving anti-VEGF treatment. These patients were eligible for treatment with EGFR inhibitors and treated, achieving a clinical benefit. However, these results were reported in a small sample size, and the evidence of the clinical benefit with EGFR inhibitors was limited to one treatment [2].

The aim of this study was to prospectively assess the *RAS* genotype in patients with primary mCRC and mutated *RAS* in solid biopsy using Idylla™ system (before first-line treatment) and disease progression after first-line treatment with FOLFOX/CAPOX + bevacizumab. *RAS* genes in liquid biopsy (before second-line treatment: panitumumab + FOLFIRI) assessed with Idylla™ system were performed. Discordant results between solid biopsy and liquid biopsy (using Idylla™ system) were confirmed by the next-generation sequencing (NGS) test (in solid and liquid biopsies).

## Materials and methods

### Patients

Patients 18 years or older, with histologically confirmed primary mCRC, *RAS* mutant clones on primary tumor before first-line initiation, at least 1 lesion with ≥ 10 mm (according to RECIST criteria), ECOG performance status 0–2, who received FOLFOX/CAPOX + bevacizumab treatment (including patients who had discontinued oxaliplatin from the FOLFOX/CAPOX treatment due to neurotoxicity) and had a liquid biopsy prior to second-line initiation were included.

### Study design

This study belonged to a phase II, multicenter, and single-arm clinical trial (2017-003242-25). It was performed in accordance with the Declaration of Helsinki, approved by the local ethics committees, and all patients gave their consent to participate.

Patients with *RAS* mutant mCRC in solid biopsy (before first-line treatment) were selected. The presence of *RAS* mutant clones was analyzed before second-line treatment in liquid biopsy.

### Solid *RAS* mutations analyses

Analysis of *RAS* tissue point mutations before first-line treatment using Idylla™ system was performed in each center. The minor allele fraction (MAF) (Amplicon-sequence tissue) with this test is 5%.

### Plasma ctDNA *RAS* mutations analyses

The analyses were done at the Complexo Hospitalario Universitario A Coruña (CHUAC) (Spain) using Idylla™ system (Biocartis, Mechelen, Belgium).

Differences in point mutations between solid biopsy (before first-line treatment) and liquid biopsy (before second-line treatment) with Idylla™ system were assessed using the NGS test (in solid and liquid biopsies) (VHIO Custom Amplicon-seq panel [6], at Vall d’Hebron Institute of Oncology (VHIO, Barcelona). The minimum variant allele frequency (MAF) was 3% for tissue samples and 1% for plasma samples.

### Statistical analysis

Mean and standard deviation (SD) for continuous variables, and frequencies and percentages for categorical variables were obtained. Analyses were performed using SAS version 9.4.

## Results

Twenty-three patients with primary mCRC and *RAS* mutation (on solid biopsy before first-line treatment) were screened in eight centers. Baseline characteristics are described in Table 1.

**Table 1:** Baseline characteristics (screened patients)

	*N* = 23
Age (years), mean (SD)	66.1 (9.8)
Males, *N* (%)	16 (69.6%)
Caucasian, *N* (%)	23 (100%)
Median time since diagnosis in months, (range)	11.1 (3.9, 42.5)
Median time since metastatic disease to baseline in months, (range)	11.1 (6.3, 13.7)
TNM stage, *N* (%)^a^	
IIIB	1 (4.3%)
IV_A_	14 (60.9%)
IV_B_	7 (30.4%)
Histology grade, *N* (%)^b^	
1	3 (13.0%)
2	8 (34.8%)
3	6 (26.1%)
Primary tumor site, *N* (%)	
Colon	21 (91.3%)
Rectum	2 (8.7%)
Tumor sidedness	
Left	14 (60.9%)
Right	8 (34.8%)
Missing	1 (4.3%)
Prior chemotherapy therapy for mCRC (first-line FOLFOX/CAPOX + bevacizumab), *N* (%)	23 (100.0%)
Radiotherapy, *N* (%)	1 (4.3%)
Prior surgery, *N* (%)	13 (56.5%)

*N* number of patients, *SD* standard deviation

^a^Missing data for one patient

^b^Missing data for six patients

Among these screened patients, none met the selection criteria (19 patients do not meet the undetectable *RAS* mutant clones in liquid biopsy prior to second-line initiation, one did not have disease progression, one was on third-line treatment, one had interstitial pneumonitis, and one received not permitted medication).

**Fig. 1:**
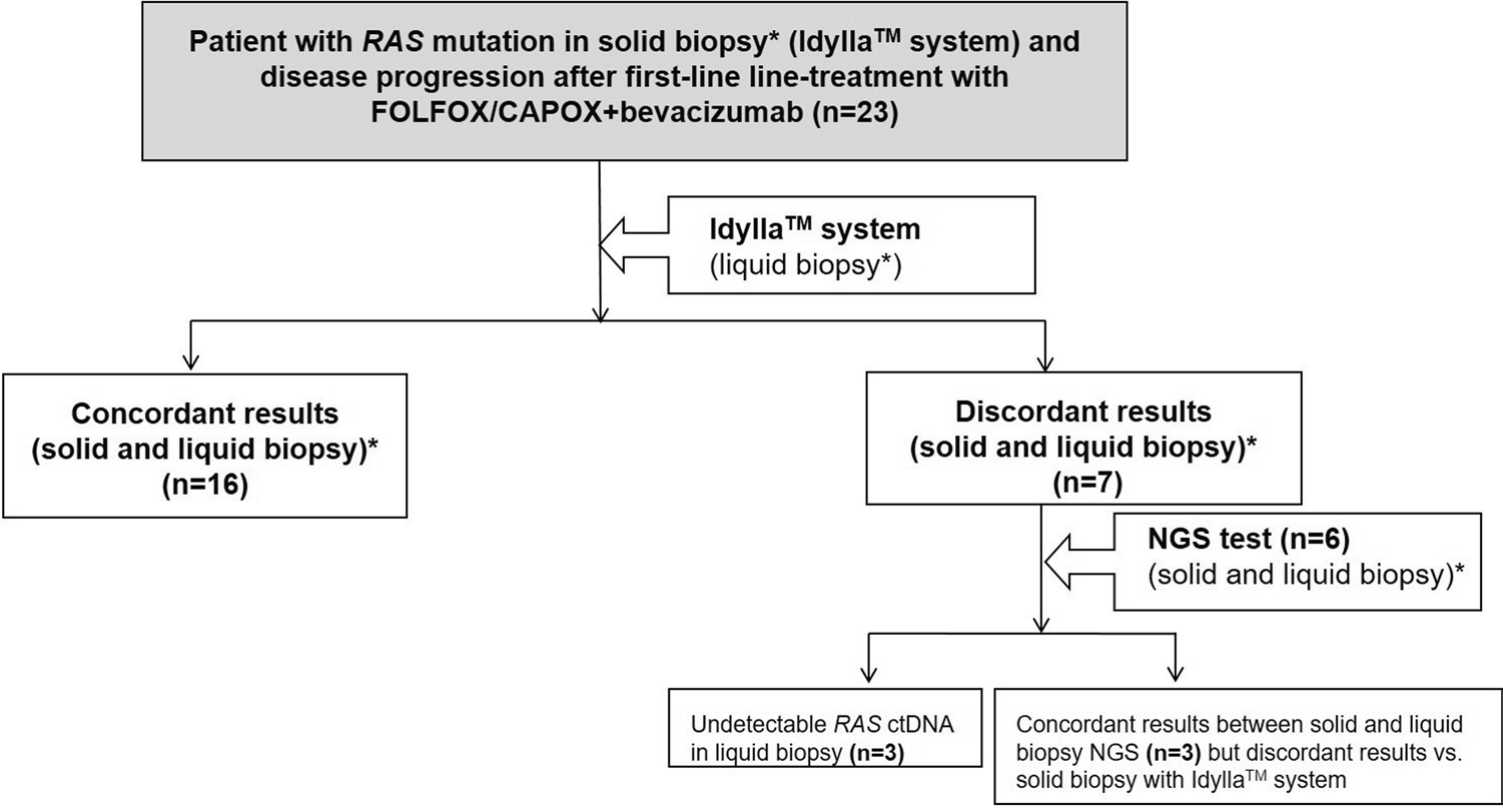
*RAS* genetic results of 23 patients with *RAS* mutant clones in solid biopsy (before first-line treatment). *Solid biopsy (before first-line treatment) and liquid biopsy (before second-line treatment). *NGS* next-generation sequencing

Although the study could not be performed, the results of *RAS* mutation analysis before first-line and second-line treatment, respectively, have reported new knowledge and learning about it (Fig. 1). *RAS* mutations were maintained before second-line treatment in 16 out of 23 (69.6%) patients (Idylla™ system) (Table 2). By contrast, some discrepancies between solid biopsy and liquid biopsy using Idylla™ system (seven out of 23 patients) were observed and were confirmed by the NGS test (Table 3). In four patients (1-003, 1-005, 1-007 and 4-001), *RAS* mutation in solid biopsy was not detected in liquid biopsy using Idylla™ system. Results in liquid biopsy using NGS test allowed to detect *KRAS* mutation in patient 1-003. Therefore, in three out of 23 patients (13.0%) (1-005, 1-007 and 4-001), undetectable ctDNA in liquid biopsy was verified by NGS test. In addition, in three patients (4-001, 3-002 and 2-002), some discrepancies in the *RAS* mutation genes (*KRAS* and *NRAS* genes) (Idylla™ system) were reported between solid and liquid biopsies. In these three patients, the results of NGS test confirmed some discrepancies vs. Idylla™ system, in solid biopsies: patient 4-001 reported *NRAS* mutation in solid biopsy using Idylla™ system when only *BRAF* mutation was confirmed by NGS test, patient 3-002 reported *KRAS* mutation in solid biopsy while it was confirmed by NGS test that this was a *NRAS* mutation. The patient 2-002 reported *NRAS* mutation in solid biopsy but it was confirmed by NGS test that this was a *KRAS* mutation. These analyses were repeated with Idylla™ system in the original tissue and corroborated that the initial results of solid biopsies with Idylla™ system had some mistakes.

**Table 2:** Patients with *RAS-*mutated mCRC with concordant results in *RAS* mutations with Idylla™ system (among solid and liquid biopsies)

Patient ID	Tissue point mutations (before first-line treatment)	Plasma point mutations (before second-line treatment)
1-001	KRAS Codon 12 (exon 2) G12C	KRAS Codon 12 (exon 2) G12C
1-002	KRAS Codon 12 (exon 2) G12D	KRAS Codon 12 (exon 2) G12D
1-004	KRAS Codon 12 (exon 2) G12C	KRAS Codon 12 (exon 2) G12C
1-006	KRAS Codon 12 (exon 2) G12C	KRAS Codon 12 (exon 2) G12C
2-001	KRAS Codon 61 (exon 3) Q61H	KRAS Codon 61 (exon 3) Q61H
2-003	KRAS Codon 13 (exon 2) G13D	KRAS Codon 13 (exon 2) G13D
2-004	KRAS Codon 12 (exon 2) G12D	KRAS Codon 12 (exon 2) G12D
3-001	KRAS Codon 12 (exon 2) G12D KRAS Codon 13 (exon 2) G13D	KRAS Codon 12 (exon 2) G12D KRAS Codon 13 (exon 2) G13D
4-002	KRAS Codon 12 (exon 2) G12D	KRAS Codon 12 (exon 2) G12D
5-001	KRAS Codon 12 (exon 2) G12V	KRAS Codon 12 (exon 2) G12V
5-002	KRAS Codon 13 (exon 2) G13D	KRAS Codon 13 (exon 2) G13D
5-003	KRAS Codon 13 (exon 2) G13D	KRAS Codon 13 (exon 2) G13D
5-004	NRAS Codon 61 (exon 3) Q61R	NRAS Codon 61 (exon 3) Q61R
5-005	KRAS Codon 12 (exon 2) G12D	KRAS Codon 12 (exon 2) G12D
6-001	KRAS Codon 12 (exon 2) G12C	KRAS Codon 12 (exon 2) G12C
6-002	KRAS Codon 117 (exon 4) K117N	KRAS Codon 117 (exon 4) K117N

**Table 3:** Patients with *RAS*-mutated mCRC with discordant results in *RAS* mutations with Idylla™ system (among solid and liquid biopsies)

Patient ID	Location of metastasis	Tissue point mutation (before first-line treatment) Idylla™ system	Tissue point mutation (before first-line treatment) NGS test	Plasma point mutation (before second-line treatment) Idylla™ system	Plasma point mutation (before second-line treatment) NGS test
1-003	lung	*KRAS* (exon 3) A59E	*KRAS* (exon 2) G12V *KRAS* (exon 3) Q61H	Not detectable	*KRAS* (exon 3) Q61H*
1-005	lung, liver	*KRAS* (exon 2) G12D	*KRAS* (exon 2) G12D	Not detectable	Not detectable
1-007	renal artery, terminal ileum	*KRAS* (exon 2) G12D	*KRAS* (exon 2) G12D	Not detectable	Not detectable
4-001	bone, left paracolic gutter	*NRAS* (exon 2) G12D	*BRAF* (exon 15) V600E	*BRAF* (exon 15) V600E/D	Not detectable
3-002	liver, lymph nodes	*KRAS* (exon 3) A59E *KRAS* (exon 3) Q61R/L	*NRAS* (exon 3) Q61R	*NRAS* (exon 3) Q61R/K	*NRAS* (exon 3) Q61R
2-002	liver, lymph nodes	*NRAS* (exon 2) G12D	*KRAS* (exon 2) G12D	*KRAS* (exon 2) G12D	*KRAS* (exon 2) G12D
2-005	liver, lymph nodes	*KRAS* (exon 3) Q61R	ND	*KRAS* (exon 3) Q61R/K	ND

*ND* not done because there was not enough sample

*0.3%

## Discussion

The genetic analysis of *RAS* genes of the screening patients that presented discordant results between solid and liquid biopsies (before first and second-line treatment, respectively) using Idylla™ system has allowed us to find some concerns that provide new evidence relevant in clinical practice.

The results of this study showed that most patients (16 patients, 70%) had concordance between solid (Gold Standard) and liquid biopsy results in *RAS *mutant clones using Idylla™ system. However, seven patients presented discordant results which were the reason to consider that the Gold Standard fails in the centers. Four of them had absence of any *RAS* mutations in plasma before initiation of second-line treatment. In three out of four, NGS test in plasma was also non-detectable. However, in the remaining patient (1-003), *KRAS* mutant could be detected in plasma with NGS test. This fact reinforces the higher sensitivity of NGS test vs. Idylla™ system. The patient’s sample that reported undetectable ctDNA in liquid biopsy with Idylla™ system presented 0.3% MAF.

Among the three patients with undetectable ctDNA, patient 1-007 was a patient who was still alive with low burden disease (2 cm lymphadenopathy: renal artery and terminal ileum) and patient 4-001 had metastasis in bone and paracolic gutter. By contrast, the patient 1-005 presented metastasis in lung and liver. The biological characteristic of the tumor is still poorly understood. Kagawa et al. [7] reported some discordances between plasma and tissues-based analyses related to the location of primary tumor, diameter, and number of metastatic lesions. Some studies reported a low proportion of undetectable *RAS* mutant clones (1.6–8.5%) in mCRC patients [8], while others found 45% (five out of 11) patients with *RAS* mutation not detected in plasma [2].

In the remaining three patients, some *RAS* mutations differences in solid biopsy using Idylla™ system and NGS test were observed. The genetic analyses were repeated a second time by the Idylla™ system in solid biopsy and support those obtained with the NGS test, which confirms that the previous results in Idylla™ system were most likely due to technical issues during routine testing. With the development of *KRAS* inhibitors, it is crucial to have an accurate result to know which codon and exon are mutated for a better therapeutic approach.

Colorectal cancer harbors a considerable heterogeneity, and the treatments imposed evolutionary pressure in selection of *RAS* mutations at progression of disease [9–11]. Genotyping cancer is mandatory in clinical practice to personalize the treatments. For example, recent evidence supports the use of the mismatch repair gene (*MMR*) testing for the implication of adjuvant therapy in patients with stage II colorectal cancer. The favorable prognosis of patients with stage II MSI-H colorectal cancer and the lack of benefit from adjuvant 5-fluorouracil-based therapy, indicate that these patients should avoid adjuvant chemotherapy. Therefore, testing for *MMR* status by MSI analysis or immunohistochemistry should be recommended in stage II colorectal cancer in patients who are candidates to adjuvant treatment is a consideration [12]. Therefore, improvement of good clinical practice to detect *RAS* mutation (especially using Idylla™ in solid biopsy), quality standard of care, monitorization, and the availability and the use of CEN technical documents and ISO standards for analytical procedures are needed.

This study has some limitations. This study was initially not designed to assess the genetic analysis of *RAS* genes in mCRC patients and the sample size was very small. The lack accessibility to liquid biopsies before first-line treatment is also an important limitation. To achieve an appropriate design, assessing *RAS* status in both solid and liquid biopsies should have been performed before first-line and before second-line treatment using both Idylla™ and NGS tests. In addition, the absence of detectable *RAS* mutations in plasma found before second-line treatment cannot certainly exclude that a *RAS* mutation might be present in the sample below the assay limit of detection. The sensitivity of the genetic analyses is relevant, and it is known that below 1% MAF, Idylla [13] has a reduced *KRAS* mutation detection in plasma.

## Conclusion

Genotyping mCRC is crucial for personalized treatments. Our results showed some discrepancies between solid and liquid biopsies. Moreover, a lower percentage of undetectable *RAS* mutant clones compared with previous studies was observed. Therefore, there is a need for clinical improvement in the accuracy of the genotype analysis, especially in solid biopsies. As there is clonal selection, this type of approach should be implemented as part of the care of these patients, which will allow an adequate follow-up. In addition, this approach will not only be implemented in mCRC but also in localized disease.
